# Real‐world effectiveness of lenvatinib monotherapy in previously treated unresectable hepatocellular carcinoma in US clinical practice

**DOI:** 10.1002/cnr2.1679

**Published:** 2022-07-13

**Authors:** Amit G. Singal, Saurabh P. Nagar, Abby Hitchens, Keith L. Davis, Shrividya Iyer

**Affiliations:** ^1^ Department of Internal Medicine UT Southwestern Medical Center Dallas Texas USA; ^2^ RTI Health Solutions Research Triangle Park North Carolina USA; ^3^ Eisai Inc. Woodcliff Lake New Jersey USA

**Keywords:** chart review, hepatocellular carcinoma, lenvatinib monotherapy, real‐world data, survival analysis

## Abstract

**Background:**

Lenvatinib monotherapy was approved in the United States for first‐line treatment of patients with unresectable hepatocellular carcinoma (uHCC) in 2018. This study assessed real‐world treatment patterns and outcomes of lenvatinib beyond first‐line systemic treatment in the United States.

**Methods:**

A retrospective study was conducted among US adults (≥18 years) with uHCC. Eligible patients initiated lenvatinib monotherapy as second‐ or later‐line systemic therapy (2L‐plus) from August 2018 to September 2019. Clinical outcomes included physician‐reported best response, progression‐free survival (PFS), and overall survival (OS).

**Results:**

Of 164 patients who received lenvatinib in 2L‐plus, most (*n* = 133; 81.1%) received lenvatinib in 2 L. There were 109 patients (66.4%) who initiated lenvatinib after immunotherapy. At lenvatinib initiation, only 31.1% of patients had Child‐Pugh class A, while half (49.4%) had Child‐Pugh class B. Most patients had Barcelona Clinic Liver Cancer stage B (23.8%) or C (38.4%) uHCC. Median duration of lenvatinib treatment was 6.9 months, with 42.7% of patients still on treatment at the end of follow‐up. Physician‐reported best response was complete and partial response for 8.5% and 44.5% of patients, respectively. PFS and OS rate estimates from lenvatinib initiation at 12 months were 51.7% and 57.8%, respectively. Among patients treated after immunotherapy, complete and partial responses were 10.1% and 43.1%, respectively, and PFS and OS estimates from lenvatinib initiation at 12 months were 52.8% and 60.0%, respectively.

**Conclusion:**

This retrospective study suggests clinical effectiveness of lenvatinib monotherapy in a real‐world setting among previously treated patients with uHCC, including among those previously treated with immunotherapy.

## 1 INTRODUCTION

Liver cancer is the fourth most common cause of cancer‐related deaths globally and accounted for approximately 30 160 deaths in the United States in 2020.^1^, ^2^ Although curative surgical options are available for patients diagnosed at an early stage, most patients continue to be diagnosed at later stages with more advanced tumor burden. In recent years, the treatment landscape for unresectable hepatocellular carcinoma (uHCC) has been rapidly evolving, with additional therapies on the horizon.

For many years, sorafenib was the only approved systemic treatment for uHCC in the United States.^3^ Lenvatinib, an oral multikinase inhibitor, was then approved as another first‐line treatment for uHCC in August 2018, based on results from the phase 3 REFLECT trial.^4^ Other tyrosine kinase inhibitors (TKIs) and immunotherapies were also approved as second‐line treatment options in uHCC. Regorafenib was the first TKI approved for second‐line treatment,^5^, ^6^ followed by approvals of cabozantinib and ramucirumab shortly thereafter.^7^ In May 2020, the combination of the immune checkpoint inhibitor atezolizumab and the antivascular endothelial growth factor (VEGF) antibody bevacizumab (atezolizumab + bevacizumab) was approved as a first‐line treatment for uHCC, demonstrating superior progression‐free survival (PFS) and overall survival (OS) compared with sorafenib.^8^ Given the rapidly evolving treatment landscape of uHCC, all second‐line treatment options have, to date, been evaluated prior to atezolizumab + bevacizumab approval, so the efficacy of TKI therapies after atezolizumab + bevacizumab approval is not well described.

Due to these recent changes in the treatment landscape, investigating lenvatinib treatment patterns and related outcomes when used later in the uHCC treatment sequence (i.e., in second or later line), particularly after treatment with immune checkpoint inhibitors in the first‐line setting, is critical to informing sequencing strategies. In this retrospective real‐world data study, we report treatment characteristics and outcomes when used as a second‐ or later‐line systemic therapy among a US patient population.

## 2 MATERIALS AND METHODS

### 2.1 Study design

This retrospective cohort study used a review of patient medical records to examine treatment patterns and outcomes among patients who received lenvatinib as a second‐ or later‐line treatment for uHCC during routine medical care. Academic‐ and community practice‐based physicians (*n* = 52) participating in the study identified eligible patients and captured detailed information using a web‐based data collection form. Physicians were eligible for participation if they had treated at least two patients with uHCC with lenvatinib in August 2018 or later, were specialized in oncology or hepatology, and were responsible for making treatment decisions for patients with uHCC under their care. All patients’ data were deidentified, and RTI International's institutional review board approved the study (STUDY00020934).

### 2.2 Patient selection

Each physician participant randomly selected three eligible patients. To minimize selection bias, physician participants who had more than three eligible patients randomly selected a letter from A to Z and selected patients whose first letter of their last names matched the randomly selected letter. Eligible patients were aged 18 years or older, had a confirmed diagnosis of uHCC, and had an Eastern Cooperative Oncology Group performance status (ECOG‐PS) of 0 or 1. All patients included in the study were treated with lenvatinib monotherapy in second or later line between August 2018 and September 2019. Patients were included in the study regardless of whether they were alive or deceased during the medical record review. Patients were excluded if they had a history of liver transplant or any other malignancy, with evidence of any active disease within 3 years of initiating lenvatinib treatment.

### 2.3 Study variables and endpoints

#### 2.3.1 Patient and physician characteristics

Physician participants reported the number of patients with uHCC they treated in the 12 months prior to data collection, the number of years they had been in practice, their medical specialty, and their primary practice setting (e.g., academic, community, or transplant center). Demographic, clinical, and treatment characteristics were extracted from patients' medical records. Demographic characteristics included patients' age at lenvatinib initiation, sex, and race/ethnicity. Clinical characteristics included patients' liver disease etiology (i.e., hepatitis B, hepatitis C, alcohol‐related steatohepatitis, or nonalcoholic steatohepatitis), cirrhosis severity (Child‐Pugh score), Charlson Comorbidity Index, ECOG‐PS, and tumor stage (Barcelona Clinical Liver Cancer [BCLC] stage). Treatment characteristics included patients' receipt of treatments or procedures prior to and after lenvatinib initiation as well as lenvatinib treatment characteristics, such as start and end dates, initial and last dose, and dose modifications (if any).

#### 2.3.2 Clinical outcomes

Dates of disease progression and death were collected from patients' medical records (if event occurred). The clinical data collected from patients' medical records also included physician‐reported best response (i.e., complete response, partial response, stable disease, or progressive disease) and the physician‐reported criteria (e.g., Response Evaluation Criteria in Solid Tumors [RECIST] version 1.1, modified RECIST [mRECIST], or physician assessment) used to evaluate best clinical response.

PFS was defined as time from lenvatinib initiation to clinical progression or death during lenvatinib treatment. OS was defined as time from lenvatinib initiation to death. For PFS, patients who did not progress during lenvatinib treatment were censored at the lenvatinib stop date. For OS, patients who were still alive at the time of data collection were censored at the date of their last available medical record.

### 2.4 Statistical analysis

This study was primarily descriptive, and no formal hypothesis testing or comparative analyses were conducted. Due to the descriptive nature of the study, the sample size was based on available resources and did not involve a formal statistical power calculation. Descriptive statistics were reported for patient and physician characteristics. All analyses were conducted in the overall cohort as well as the subgroups of patients who received lenvatinib in second line only, in third or later lines, after receiving immunotherapy, and after receiving atezolizumab + bevacizumab. Clinical outcomes were described overall, stratified by BCLC stage and Child‐Pugh class as well for each subgroup, and chi‐square analysis was used to assess differences in best clinical response between patients who were assessed using RECIST 1.1 and patients who were assessed using mRECIST. Time‐to‐event outcomes (i.e., PFS and OS) were estimated using the Kaplan–Meier method to account for right censoring. PFS and OS rate estimates at 3, 6, 9, and 12 months were reported. All analyses were conducted using SAS statistical software (SAS. Version 9.4. Cary, NC: SAS Institute Inc.; 2012).

## 3 RESULTS

### 3.1 Physician characteristics

Most (94.2%) of the 52 physician participants who treated patients with lenvatinib in second or later line were medical oncologists. Overall, they had been in practice for a median of 15 years and had seen a median of 50 patients with HCC in the prior 12 months. Most of the physician participants reported that they primarily practiced in a cancer center or tertiary referral treatment center (36.5%), or a private hospital or clinic (32.7%). A quarter (25%) reported other academic or teaching hospital as their primary practice setting, and a few (5.8%) reported other nonteaching hospital as their primary practice setting.

### 3.2 Patient characteristics

Patient characteristics for the overall cohort (*n* = 164), the subgroup of patients treated with lenvatinib in second line only (*n* = 133), and the subgroup of patients treated with lenvatinib after receiving immunotherapy (*n* = 109) are shown in Table 1. The two subgroups are not mutually exclusive. Patients in the overall cohort had a median age of 61.6 years at lenvatinib initiation, and most identified as male (72%) and White (55.5%) or African American (23.2%). Most patients had signs of liver dysfunction, with 49.4% having Child‐Pugh class B and 11.6% having Child‐Pugh class C. The most frequent etiology ascertained from patients' medical records was alcohol‐related liver disease (31.7%), followed by hepatitis C (26.2%), nonalcoholic steatohepatitis (21.3%), and hepatitis B (14.6%). Over a third of patients (38.4%) were BCLC stage C, followed by stage B (23.8%), stage D (12.2%), and stage 0 or A (11.6%). Portal vein invasion was reported in 11.6% of patients, although only one patient had main portal vein invasion. Approximately half (55.5%) of patients remained alive at the end of study follow‐up (median follow‐up approximately 17 months).

**TABLE 1:** Patient demographic and clinical characteristics

Characteristic	Patients treated with lenvatinib in second or later line (*N* = 164)	Patients treated with lenvatinib in second line only (*n* = 133)	Patients treated with lenvatinib postimmunotherapy (*n* = 109)	Patients treated with lenvatinib postatezolizumab + bevacizumab (*n* = 39)
*Demographic characteristics*				
Age, years				
Mean (SD)	60.8 (7.8)	61.1 (7.6)	60.7 (7.8)	60.3 (8.8)
Median (Q1, Q3)	61.6 (55.6, 66.0)	61.5 (56.3, 65.8)	62.1 (55.1, 66.1)	61.2 (54.7, 67.7)
Sex, *n* (%)				
Male	118 (72)	94 (70.7)	78 (71.6)	26 (66.7)
Race/ethnicity, *n* (%)				
White	91 (55.5)	76 (57.1)	55 (50.5)	19 (48.7)
African American	38 (23.2)	29 (21.8)	27 (24.8)	10 (25.6)
Hispanic/Latino	14 (8.5)	10 (7.5)	10 (9.2)	3 (7.7)
Asian	19 (11.6)	16 (12)	15 (13.8)	7 (17.9)
Middle Eastern	2 (1.2)	2 (1.5)	2 (1.8)	0 (0)
Not reported	0 (0)	0 (0.0)	0 (0)	0 (0)
Duration of follow‐up from initial HCC date, months ^a^				
Mean (SD)	18.2 (7.5)	17.2 (7.3)	18.6 (7)	16.4 (4.8)
Median (Q1, QR)	17.8 (12.8, 21.8)	17.4 (12.0, 20.7)	18 (13.7, 22.0)	16.3 (12.6, 18.8)
Vital status at end of follow‐up, *n* (%)^a^				
Deceased	72 (43.9)	57 (42.9)	51 (46.8)	19 (48.7)
Death related to HCC	65 (90.3)	52 (91.2)	48 (94.1)	18 (94.7)
Death unrelated to HCC	7 (9.7)	5 (8.8)	3 (5.9)	1 (5.3)
Alive	91 (55.5)	75 (56.4)	57 (52.3)	19 (48.7)
*Clinical characteristics*				
Weight, kg				
Mean (SD)	78.1 (15.1)	78.6 (15.7)	76.1 (15.4)	75.5 (15.5)
< 60, *n* (%)	10 (8.5)	10 (10)	9 (12.2)	4 (14.8)
≥ 60, *n* (%)	108 (91.5)	90 (90)	65 (87.8)	23 (85.2)
ECOG score, *n* (%)				
0	48 (29.3)	44 (33.1)	40 (36.7)	15 (38.4)
1	115 (70.1)	88 (66.2)	68 (62.4)	23 (58.9)
Not reported	1 (0.6)	1 (0.7)	1 (0.9)	1 (2.6)
BCLC stage, *n* (%)				
0 or A	19 (11.6)	17 (12.8)	14 (12.8)	5 (12.8)
B	39 (23.8)	29 (21.8)	29 (26.6)	6 (15.4)
C	63 (38.4)	53 (39.9)	35 (32.1)	17 (43.6)
D	20 (12.2)	13 (9.8)	16 (14.7)	3 (7.7)
Unknown	23 (14)	21 (15.8)	15 (13.8)	8 (20.5)
Child‐Pugh class, *n* (%)				
A	51 (31.1)	41 (30.8)	35 (32.1)	10 (25.6)
B	81 (49.4)	67 (50.4)	46 (42.2)	19 (48.7)
C	19 (11.6)	12 (9)	16 (14.7)	3 (7.7)
Unknown	13 (7.9)	13 (9.8)	12 (11)	7 (18.0)
Etiology, *n* (%)				
Hepatitis B	24 (14.6)	23 (17.3)	11 (10.1)	3 (7.7)
Hepatitis C	43 (26.2)	28 (21.1)	31 (28.4)	8 (20.5)
Alcohol‐related disease	52 (31.7)	40 (30.1)	31 (28.4)	13 (33.3)
Nonalcoholic steatohepatitis	35 (21.3)	29 (21.8)	22 (20.2)	7 (18.0)
Presence of portal thrombosis, *n* (%)	19 (11.6)	11 (8.3)	12 (11)	2 (5.1)
VP0	1 (5.3)	1 (9.1)	1 (8.3)	0 (0)
VP1	6 (31.6)	5 (45.5)	2 (16.7)	0
VP2	2 (10.5)	2 (18.2)	1 (8.3)	0
VP3	0 (0)	0 (0)	0 (0)	0
VP4	1 (5.3)	1 (9.1)	1 (8.3)	1 (50.0)
Grade not reported	9 (47.4)	2 (18.2)	7 (58.3)	1 (50.0)
Prior procedure, *n* (%)				
Transarterial chemoembolization	23 (14)	19 (14.3)	11 (10.1)	3 (7.7)
Radiofrequency ablation	9 (5.5)	9 (6.8)	5 (4.6)	1 (2.6)
Other	3 (1.8)	3 (2.3)	0 (0)	0
Alpha‐fetoprotein level				
< 200 ng/ml	55 (33.5)	44 (33.1)	35 (32.1)	11 (28.2)
≥ 200 ng/ml	57 (34.8)	43 (32.3)	34 (31.2)	10 (25.4)
Not reported	52 (31.7)	46 (34.6)	40 (36.7)	18 (46.2)

Abbreviations: BCLC, Barcelona Clinical Liver Cancer; ECOG, Eastern Cooperative Oncology Group; HCC, hepatocellular carcinoma; SD, standard deviation.

^a^ Last available follow‐up was defined as the earliest of death, last medical record entry date, or February 1, 2020.

### 3.3 Lenvatinib treatment characteristics

For patients who received lenvatinib in second or later line, the most frequent first‐line therapy reported was sorafenib (45.7%), followed by atezolizumab + bevacizumab (20.1%), pembrolizumab (18.9%), and nivolumab (8.5%). The median duration of patients' first‐line treatment was 5.1 months **(**Table 2 **)**. Sorafenib and atezolizumab + bevacizumab remained the 2 most common first‐line therapies in the subgroup with Child‐Pugh class A (47.1% and 17.7%, respectively) and those with Child‐Pugh class B (50.6% and 18.5%, respectively) at lenvatinib initiation.

**TABLE 2:** Lenvatinib treatment characteristics

Lenvatinib treatment characteristic	Patients treated with lenvatinib in second or later line (*N* = 164)	Patients treated with lenvatinib in second line only (*n* = 133)	Patients treated with lenvatinib postimmunotherapy (*n* = 109)	Patients treated with lenvatinib postatezolizumab + bevacizumab (*n* = 39)
Time to initiation from diagnosis of unresectable HCC, months				
Mean (SD)	9.9 (7.5)	8.7 (6.8)	9.9 (7.3)	7.6 (4.3)
Median (Q1, Q3)	8.2 (4.5, 13.8)	7.0 (4.3, 12.0)	8.0 (4.3, 13.7)	7.0 (4.2, 10.1)
First‐line treatment prior to lenvatinib, *n* (%)				
Sorafenib	75 (45.7)	49 (36.8)	25 (22.9)	4 (10.3)
Atezolizumab + bevacizumab	33 (20.1)	33 (24.8)	33 (30.3)	33 (84.6)
Pembrolizumab	31 (18.9)	29 (21.8)	31 (28.4)	0 (0)
Nivolumab	14 (8.5)	14 (10.5)	14 (12.8)	0 (0)
Other^a^	10 (6.1)	7 (5.3)	6 (5.5)	2 (5.1)
Not reported	1 (0.6)	1 (0.8)	0 (0)	0 (0)
Duration of first‐line treatment, months				
Median (Q1, Q3)	5.1 (2.8, 8.8)	4.6 (2.8, 8.3)	4.9 (2.8, 7.9)	4.3 (2.1, 7.2)
Initial lenvatinib dose, *n* (%)				
4 mg	1 (0.6)	1 (0.8)	0 (0)	0 (0)
8 mg	18 (11)	16 (12)	13 (11.9)	10 (25.6)
12 mg	87 (53.1)	68 (51.1)	54 (49.5)	16 (41.0)
24 mg	21 (12.8)	17 (12.8)	12 (11)	2 (5.1)
Other	37 (22.6)	31 (23.3)	30 (27.5)	11 (28.2)
Dose reduction during lenvatinib treatment, *n* (%)				
Yes	17 (10.4)	10 (7.5)	13 (11.9)	3 (7.7)
No	144 (87.8)	121 (91)	95 (87.2)	36 (92.3)
Not reported	3 (1.8)	2 (1.5)	1 (0.9)	0 (0)
Treatment status at end of follow‐up, *n* (%)				
Therapy discontinued and initiated next line	10 (6.1)	7 (5.3)	10 (9.2)	3 (7.7)
Therapy discontinued and did not initiate next line	84 (51.2)	69 (51.9)	61 (56)	20 (51.3)
Died	62 (73.8)	49 (71)	41 (67.2)	15 (75.0)
Lost to follow‐up	22 (26.2)	20 (29)	20 (32.8)	5 (25.0)
Lenvatinib treatment ongoing	70 (42.7)	57 (42.9)	38 (34.9)	16 (41.0)
Duration of treatment, months				
Mean (SD)	7.4 (4.1)	7.5 (4.3)	7.6 (4.3)	8.8 (5.1)
Median (Q1, Q3)	6.9 (3.7, 10.4)	6.9 (3.5, 10.4)	6.9 (3.5, 11.1)	9.0 (3.1, 12.0)
Time to next treatment, months				
Mean (SD)	9.3 (3.2)	9.4 (2.9)	9.3 (3.2)	10.3 (3.7)
Median (Q1, Q3)	8.2 (6.5, 12.1)	8.7 (6.5, 12.1)	8.2 (6.5, 12.1)	12.1 (6.0, 12.7)
Total number of subsequent lines of systemic therapy received after lenvatinib until last available follow‐up, *n* (%)^b^				
0	154 (93.9)	126 (94.7)	99 (90.8)	36 (92.3)
1	10 (6.1)	7 (5.3)	10 (9.2)	3 (7.7)
2	0 (0)	0 (0)	0 (0)	0 (0)
Common systemic therapy regimens among first subsequent line of treatment after lenvatinib, *n* (%)				
Sorafenib	2 (20)	2 (28.6)	2 (20)	0 (0)
Immunotherapy	6 (60)	3 (42.9)	6 (60)	3 (7.7)
Chemotherapy	0 (0)	0 (0)	0 (0)	0 (0)
Regorafenib	0 (0)	0 (0)	0 (0)	0 (0)
Cabozantinib	1 (10)	1 (14.3)	1 (10)	0 (0)
Interferon + vincristine	1 (10)	1 (14.3)	1 (10)	0 (0)

Abbreviations: HCC, hepatocellular carcinoma; SD, standard deviation.

^a^ Other includes carboplatin, carboplatin + vinblastine, cisplatin, cisplatin + vincristine, ipilimumab, pembrolizumab + carboplatin, pembrolizumab + regorafenib, sorafenib + nivolumab + cisplatin, and sunitinib.

^b^ Last available follow‐up was defined as the earliest occurrence of death, last medical record entry, or February 1, 2020.

The median duration of lenvatinib treatment was 6.9 months. Dose reductions were reported in 10.4% of patients, and 57.3% had discontinued lenvatinib. Of the 94 patients who discontinued lenvatinib, 62 (65.9%) died, 10 (10.6%) initiated next‐line therapy, and 22 (23.4%) were lost to follow‐up. Of the 10 patients who initiated next‐line therapy, most (60%) initiated immunotherapy. The duration of lenvatinib treatment was similar among patients with Child‐Pugh class A (median, 7.7 months) and Child‐Pugh class B (median, 6.7 months) at lenvatinib initiation, although dose reductions were higher in patients with Child‐Pugh class B than Child‐Pugh class A (12.4 vs. 7.8%). Treatment characteristics for patients who were treated with lenvatinib in second line only are shown in Table 2.

### 3.4 Clinical outcomes

#### 3.4.1 Overall response

**FIGURE 1:**
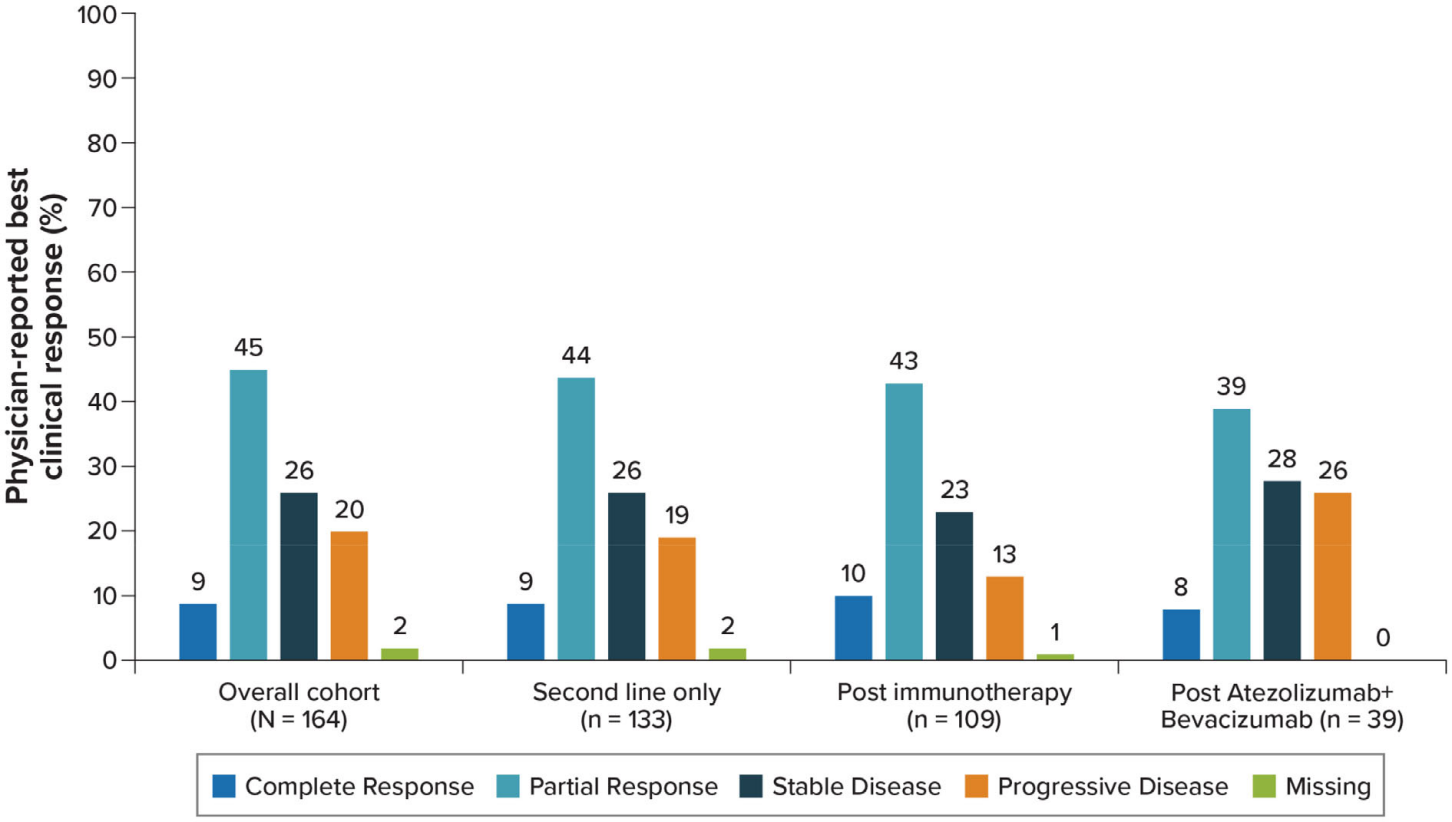
Physician‐reported best overall clinical response

Best clinical response for the overall cohort was most commonly assessed by using RECIST 1.1 (59.7%) and mRECIST (10.3%). Overall, physician‐reported best clinical response included complete response in 8.5% of patients, partial response in 44.5%, stable disease in 25.6%, and progressive disease in 19.5% (Figure 1). Best clinical response was similar between patients with Child‐Pugh class A and class B (nominal *p* = 0.11). Complete or partial response was reported for 61.2% of patients assessed with RECIST 1.1 and 35.3% of patients assessed with mRECIST. Best clinical response stratified by assessment criteria, Child‐Pugh class, and BCLC stage is shown in Table 3. In the subgroup of patients who were treated with lenvatinib in second line only (*n* = 133), physician‐reported best clinical response included complete response in 9% of patients, partial response in 44.4%, and stable disease in 25.6%.

**TABLE 3:** Physician‐reported best overall clinical response by response criteria, Child‐Pugh class, and BCLC stage

Characteristic	Physician‐reported best clinical response (complete or partial), *n* (%)
Overall (*n* = 164)	87 (53.1)
Response by criteria	
RECIST 1.1 (*n* = 98)^a^	60 (61.2)
mRECIST (*n* = 17)	6 (35.3)
Physician clinical assessment (*n* = 18)	7 (38.9)
Other (*n* = 11)	4 (36.4)
Not reported/unknown (*n* = 20)	10 (50)
Response by Child‐Pugh class	
A (*n* = 51)^b^	33 (64.7)
B (*n* = 81)	41 (50.6)
C (*n* = 19)	6 (31.6)
Unknown (*n* = 13)	7 (53.8)
Response by BCLC stage	
0 or A (*n* = 19)	14 (73.7)
B (*n* = 39)	30 (76.9)
C (*n* = 63)	28 (44.4)
D (*n* = 20)	6 (30)
Unknown (*n* = 23)	9 (39.1)

Abbreviations: BCLC, Barcelona Clinical Liver Cancer; mRECIST, modified RECIST; RECIST, Response Evaluation Criteria in Solid Tumors.

^a^ Comparison between RECIST 1.1 and mRECIST nominal *p* = .046.

^b^ Comparison between Child‐Pugh class A and B nominal *p* = .112.

#### 3.4.2 Progression‐free survival

**FIGURE 2:**
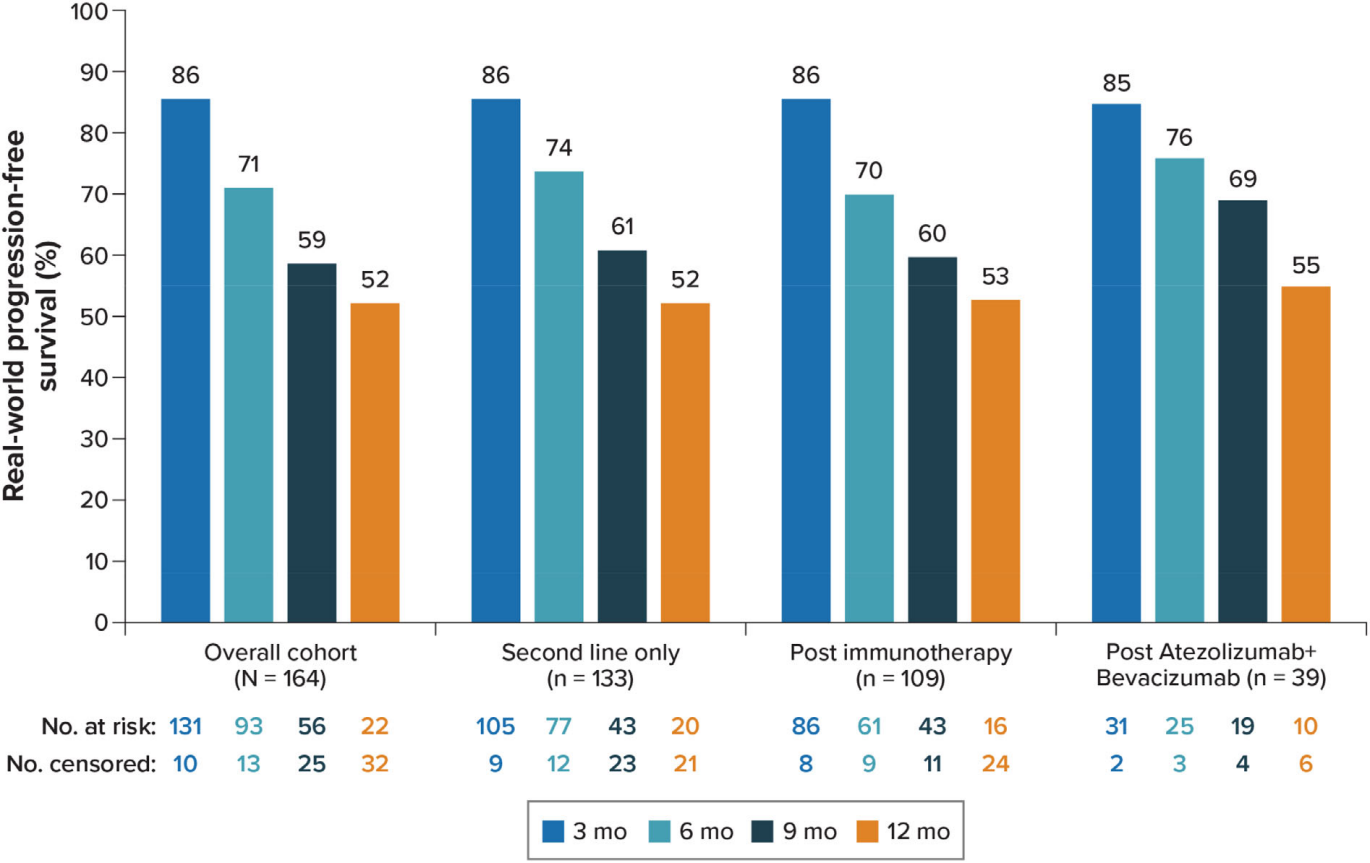
Progression‐free survival

Disease progression was determined in most patients by a combination of clinical diagnosis of progression (50.8%), radiological findings (69.2%), physical examination (36.9%), and other measures such as alpha‐fetoprotein level (9.2%). Overall, 65 patients (39.6%) had HCC progression (*n* = 55) or death (*n* = 10). The estimated median PFS from lenvatinib initiation was 12.5 months (95% confidence interval [CI] range, 10.4 months to not estimable). Estimates for PFS from lenvatinib initiation at 6 months and 12 months were 70.5% and 51.7%, respectively (Figure 2). In the subgroup of patients who initiated lenvatinib in second line only, the estimated median PFS from lenvatinib initiation was also 12.5 months (95% CI range, 10.4 months to not estimable). PFS estimates among patients who initiated lenvatinib in second line only are shown in Figure 2.

#### 3.4.3 Overall survival

**FIGURE 3:**
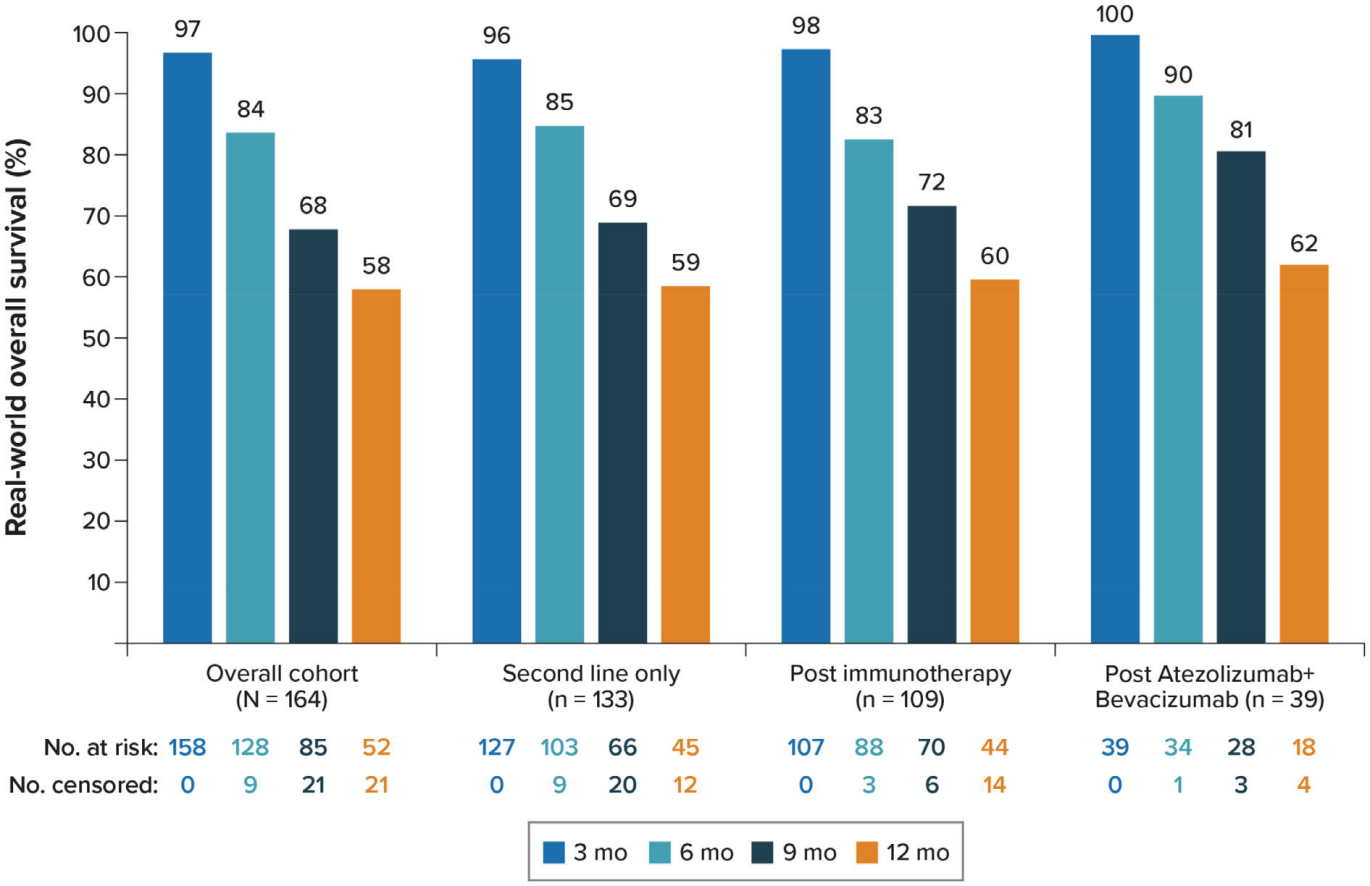
Overall survival

At the end of study follow‐up, 72 patients (43.9%) who initiated lenvatinib in second or later line were deceased. The estimated median OS was 14 months (95% CI range, 11.6–21.2 months), with OS estimates at 6 and 12 months from lenvatinib initiation of 83.9% and 57.8%, respectively (Figure 3 **)**. In the subgroup of patients who initiated lenvatinib in second line only, the estimated median OS was 14.1 months (95% CI range, 11.6 months to not estimable). OS among patients who initiated lenvatinib in second line only are shown in Figure 3.

### 3.5 Patients treated with lenvatinib postimmunotherapy

#### 3.5.1 Lenvatinib treatment characteristics

Two‐thirds (*n* = 109, 66.5%) of the overall cohort had received prior immune checkpoint inhibitor therapy before lenvatinib initiation, including 39 who had received atezolizumab + bevacizumab. The median lenvatinib starting dose among both the postimmunotherapy subgroup and the postatezolizumab + bevacizumab subgroup was 12 mg, and the median duration of lenvatinib treatment was 6.9 and 9 months, respectively. Lenvatinib dose reduction was reported in 11.9% of patients who initiated lenvatinib postimmunotherapy and 7.7% of those treated with lenvatinib after atezolizumab + bevacizumab. Many patients in both the postimmunotherapy subgroup (65.1%) and the postatezolizumab + bevacizumab subgroup (59%) had discontinued treatment by the end of study follow‐up. Of the 71 patients who discontinued lenvatinib in the postimmunotherapy subgroup, 41 (57.7%) died, 10 (14%) initiated next‐line treatment, and 20 (28.2%) were lost to follow‐up. Of the 23 patients who discontinued lenvatinib in the postatezolizumab + bevacizumab subgroup, 15 (65.2%) died, 3 (13%) initiated next‐line therapy, and 5 (21.7%) were lost to follow‐up.

#### 3.5.2 Overall response

Physician‐reported best clinical response for patients treated with lenvatinib postimmunotherapy included complete response in 10.1% of patients, partial response in 43.1%, stable disease in 22.9%, and progressive disease in 22.9%. Complete or partial response was reported for 59.0% of patients assessed with RECIST 1.1 and 40.0% of patients assessed with mRECIST. Results were similar among patients who were treated post–atezolizumab + bevacizumab, with physician‐reported complete response in 7.7%, partial response in 38.5%, stable disease in 28.2%, and progressive disease in 25.6% of patients.

#### 3.5.3 Progression‐free survival

The median PFS from lenvatinib initiation among patients who initiated lenvatinib post‐immunotherapy was 12.5 months (95% CI range, 8.3 months to not estimable). Estimates for PFS at 6 and 12 months were 69.8% and 52.8%, respectively. Results were consistent in the subgroup of patients who initiated lenvatinib postatezolizumab + bevacizumab, with a median PFS from lenvatinib initiation of 12.5 months (95% CI range, 10.4 months to not estimable) and estimates at 6 and 12 months of 76% and 55.4%, respectively **(**Figure 2; Figure S1).

#### 3.5.4 Overall survival

At the end of study follow‐up, 51 patients (46.8%) who initiated lenvatinib postimmunotherapy were deceased. The estimated median OS was 14.4 months (95% CI range, 11.6 months to not estimable), and estimates for OS rate at 6 and 12 months were 83.3% and 60%, respectively. Among patients who initiated lenvatinib postatezolizumab + bevacizumab, 19 (48.7%) were deceased at the end of study follow‐up. The median OS for this subgroup was 14 months (95% CI range, 11.6 months to not estimable), with estimates at 6 and 12 months of 89.5% and 62.2%, respectively **(**Figure 3; Figure S2).

## 4 DISCUSSION

This study was among the first to use real‐world data to examine clinical outcomes of second‐ or later‐line lenvatinib among patients in the United States. It was also the first real‐world study in the United States to examine clinical outcomes in patients initiating lenvatinib treatment after being treated with immunotherapy, including atezolizumab + bevacizumab, a group for which there are few data to guide clinical decision‐making. Overall, approximately 10% of patients were reported to require dose reductions. Lenvatinib also appeared to be effective in this setting, with best clinical response reported as complete or partial response for approximately half of all patients. Estimates of PFS and OS at 12 months were 52% and 57.8%, respectively.

Prior evaluations of lenvatinib outcomes in a real‐world setting have primarily focused on lenvatinib in extended populations when used as first‐line therapy. There have been few studies examining lenvatinib in the second‐ or later‐line therapy setting, and these studies have included heterogeneous populations with few patients who had received prior immunotherapy. A multicenter study conducted in Japan (*N* = 152) reported favorable data for patients with uHCC using lenvatinib in second‐ or later‐line therapy (95 and 57 patients, respectively), with 12‐month PFS and OS estimates of 30% and 60%, respectively.^9^ In another multicenter study from Japan including 97 patients treated with lenvatinib in the second line, median PFS ranged from 5.5 to 11.8 months and median OS ranged from 9.7 to 15.2 months, depending on BCLC stage and albumin–bilirubin score.^10^ Finally, a retrospective multicenter analysis of 17 patients in Korea treated with second‐ or later‐line lenvatinib had a median PFS of 4.1 months (95% CI range, 3.1–5.1 months) and a median OS of 6.4 months (95% CI range, 5.1–7.7 months) from lenvatinib initiation.^11^ Taken together, findings from other real‐world evaluations are consistent with our study in suggesting that lenvatinib is likely an effective treatment in second‐ or later‐line uHCC.

Currently, 3 TKIs (regorafenib, cabozantinib, and ramucirumab) have been approved for second‐line treatment of advanced HCC.^7^ Each of these TKIs was assessed in patients who had previously been treated with sorafenib but not immunotherapy.^12^, ^13^, ^14^ Therefore, it is unclear if TKIs would have the same effectiveness in patients who were previously treated with atezolizumab + bevacizumab, especially since bevacizumab acts upon the VEGF pathway. Lenvatinib acts upon a broader set of pathways, such as the fibroblast growth factor receptor (FGFR) pathway, vascular endothelial growth factor receptor (VEGFR), platelet‐derived growth factor receptor‐alpha (PDGFRα), and RET.^15^ Therefore, it may have potential to provide clinical benefit to patients treated post immunotherapy or who are not candidates for immunotherapy.^16^ Our findings corroborate this rationale and support that lenvatinib may be effective in second‐line or later treatment, with an estimated median PFS of 12.5 months and median OS from lenvatinib initiation of 14.1 months. Notably, patients treated with lenvatinib postatezolizumab + bevacizumab had similar estimated median PFS (12.5 months) and OS (14 months) to the overall second‐ or later‐line subgroup.

While not directly comparable with the results of the current study, previously reported real‐world lenvatinib treatment outcomes in the first‐line setting found that patients with Child‐Pugh class B had favorable outcomes when treated with lenvatinib, with physicians reporting objective responses in approximately 70% of patients.^15^ Given that many patients who have Child‐Pugh class A in first line progress to Child‐Pugh class B in second line, it is important to examine if patients with Child‐Pugh class B show similarly favorable outcomes when treated with lenvatinib in later lines. Our study provides additional evidence for this population. We found that approximately half of patients with Child‐Pugh class B who were treated with lenvatinib in a second‐ or later‐line setting had reported objective responses; estimated median PFS was 11.9 months (95% CI range, 7.4 months to not estimable) and overall survival was 13.5 months (95% CI range, 11.2–16.3 months) for these patients. While second‐line Child‐Pugh class B patients are common in clinical practice, there are few treatment options for this patient population. Treatment options are particularly limited if these patients have already been treated with atezolizumab + bevacizumab, which limits the utility of nivolumab. Our findings suggest that lenvatinib may be a treatment option when few are available.

This study has several limitations. The retrospective study design did not allow for the collection of safety data, and the reliance on physicians who consented to participate may contribute to self‐selection bias. Although a random patient chart selection approach was recommended to minimize bias, potential for selection bias cannot be completely ruled out. Additionally, clinical data were entered into electronic case report forms by physicians and clinical research staff, which may have resulted in inadvertent entry or keying errors and missing data. Staging and liver function scores were as reported by physicians in patient records with no requirement for reconfirmation. Clinical practices may have also varied in the frequency with which they used clinical scans, and clinical responses were determined based on physician assessment with (or without) published response criteria. Although our study included a large number of patients treated with lenvatinib in second line or later, our study was not powered to identify factors associated with treatment response. Comparisons between subgroups included within this study should not be made as differences in baseline characteristics have not been adjusted for in the current analyses. Given the small sample size in the postatezolizumab + bevacizumab subgroup, future additional research is warranted to confirm the study findings. Finally, although OS could have been affected by postprogression therapy, we were unable to evaluate the differential impact of postprogression therapies on survival owing to an insufficient number of patients for this analysis.

## 5 CONCLUSION

This retrospective cohort study provides valuable insight into real‐world lenvatinib treatment patterns and outcomes among previously treated patients with uHCC in the United States. Overall, our study reports clinical outcomes for patients with uHCC treated with lenvatinib in second‐ or later‐line treatment, and these findings are consistent in a subgroup of patients who initiated lenvatinib after immunotherapy. To our knowledge, this study is also the first to leverage real‐world data to examine clinical outcomes in patients who initiated lenvatinib after atezolizumab + bevacizumab. Therefore, this study provides much needed context for clinical decision‐making regarding this distinct patient population.

## AUTHOR CONTRIBUTIONS

Amit G. Singal, Saurabh P. Nagar, Abby Hitchens, Keith L. Davis, and Shrividya Iyer made significant contributions to this work, whether in the conception, study design, execution, acquisition of data, analysis and interpretation, or in all these areas; participated in drafting, revising, or critically reviewing the article; approved the final article; and agree to be accountable for the work.

**A.G.S.**: Conceptualization (equal); investigation (equal); supervision (equal); writing – review and editing (equal). **S.P.N.**: Conceptualization (equal); data curation (equal); formal analysis (equal); investigation (equal); methodology (equal); project administration (equal); software (equal); visualization (equal); writing – review and editing (equal). **A.H.**: Data curation (equal); formal analysis (equal); methodology (equal); project administration (equal); software (equal); validation (equal); writing – review and editing (equal). **K.L.D.**: Conceptualization (equal); formal analysis (equal); investigation (equal); methodology (equal); resources (equal); supervision (equal); writing – review and editing (equal). **S.I.**: Conceptualization (equal); funding acquisition (equal); investigation (equal); methodology (equal); project administration (equal); resources (equal); supervision (equal); writing – review and editing (equal).

## FUNDING INFORMATION

This study was funded by Eisai Inc.

## CONFLICT OF INTEREST

Keith L. Davis, Abby Hitchens, and Saurabh P. Nagar are salaried employees of RTI Health Solutions, and this study was performed under a research contract between RTI Health Solutions and Eisai Inc. Shrividya Iyer is an employee of Eisai Inc. Amit G. Singal has been on advisory boards and served as a consultant for Genentech, Bayer, Eisai, BMS, Exelixis, AstraZeneca, and TARGET RWE.

## Supporting information


**Appendix S1:** Supporting Information

## Data Availability

Research data are not shared.
