# Supplementary material for: Real‐world effectiveness of lenvatinib monotherapy in previously treated unresectable hepatocellular carcinoma in US clinical practice
Source: Cancer Rep (Hoboken). 2022 Jul 13;6(1):e1679. doi: 10.1002/cnr2.1679 (PMC9875657; doi:10.1002/cnr2.1679)

Real-World Effectiveness of Lenvatinib Monotherapy in Previously Treated Unresectable Hepatocellular Carcinoma in US Clinical Practice

Amit G. Singal, MD, UT Southwestern Medical Center
Saurabh P. Nagar, MS, RTI Health Solutions
Abby Hitchens, MPH, RTI Health Solutions
Keith L. Davis, MA, RTI Health Solutions
Shrividya Iyer, PhD, Eisai Inc.

Corresponding author:

Amit G. Singal, MD
Division of Digestive and Liver Disease
UT Southwestern Medical Center
5323 Harry Hines Blvd.
Dallas, Texas 75390-9030
Tel: 734-657-7894
Email: [amit.singal@utsouthwestern.edu](mailto:amit.singal@utsouthwestern.edu)

# Supplemental Appendix

Figure S-1. Kaplan-Meier Curve of Progression-Free Survival


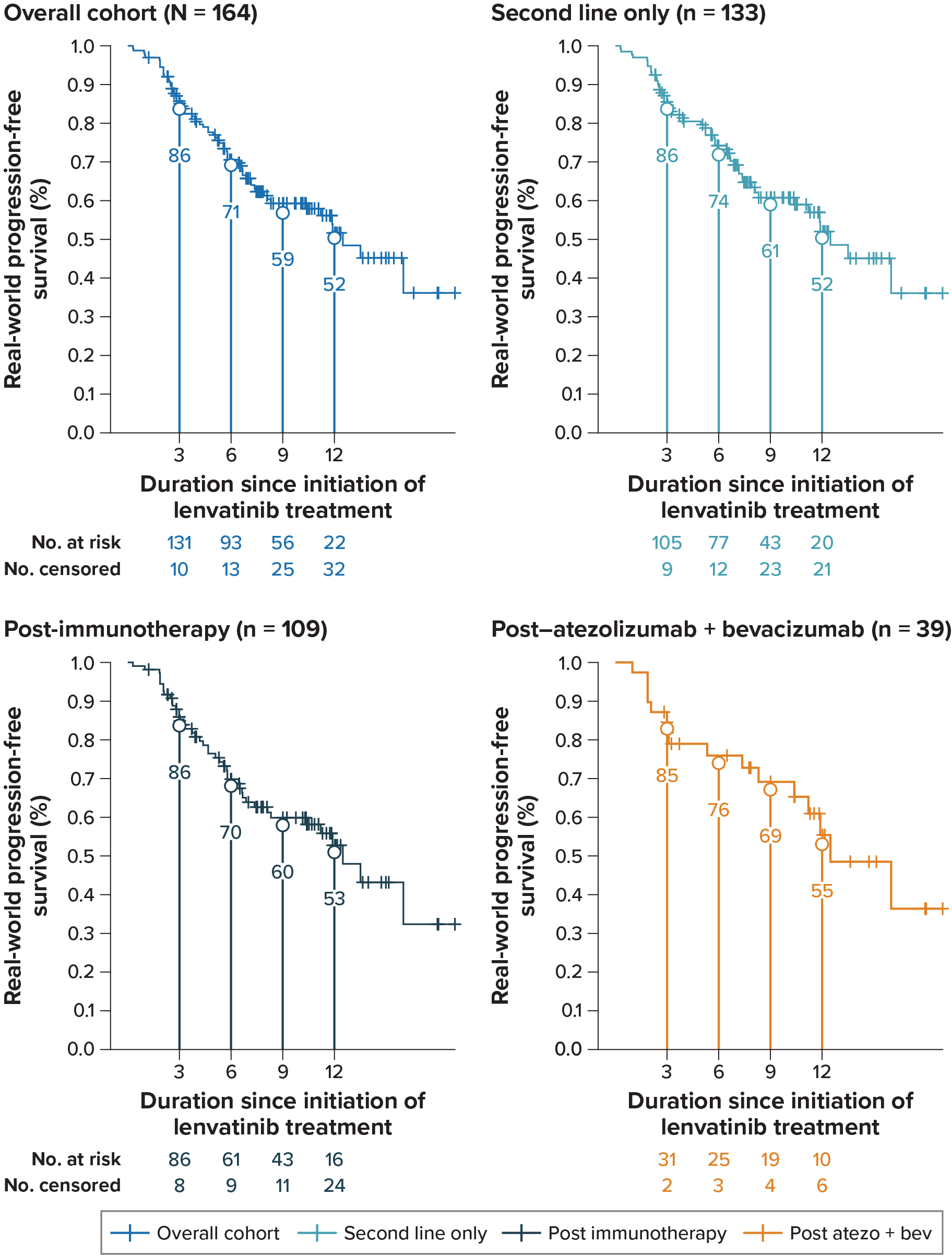


Figure S-2. Kaplan-Meier Curve of Overall Survival


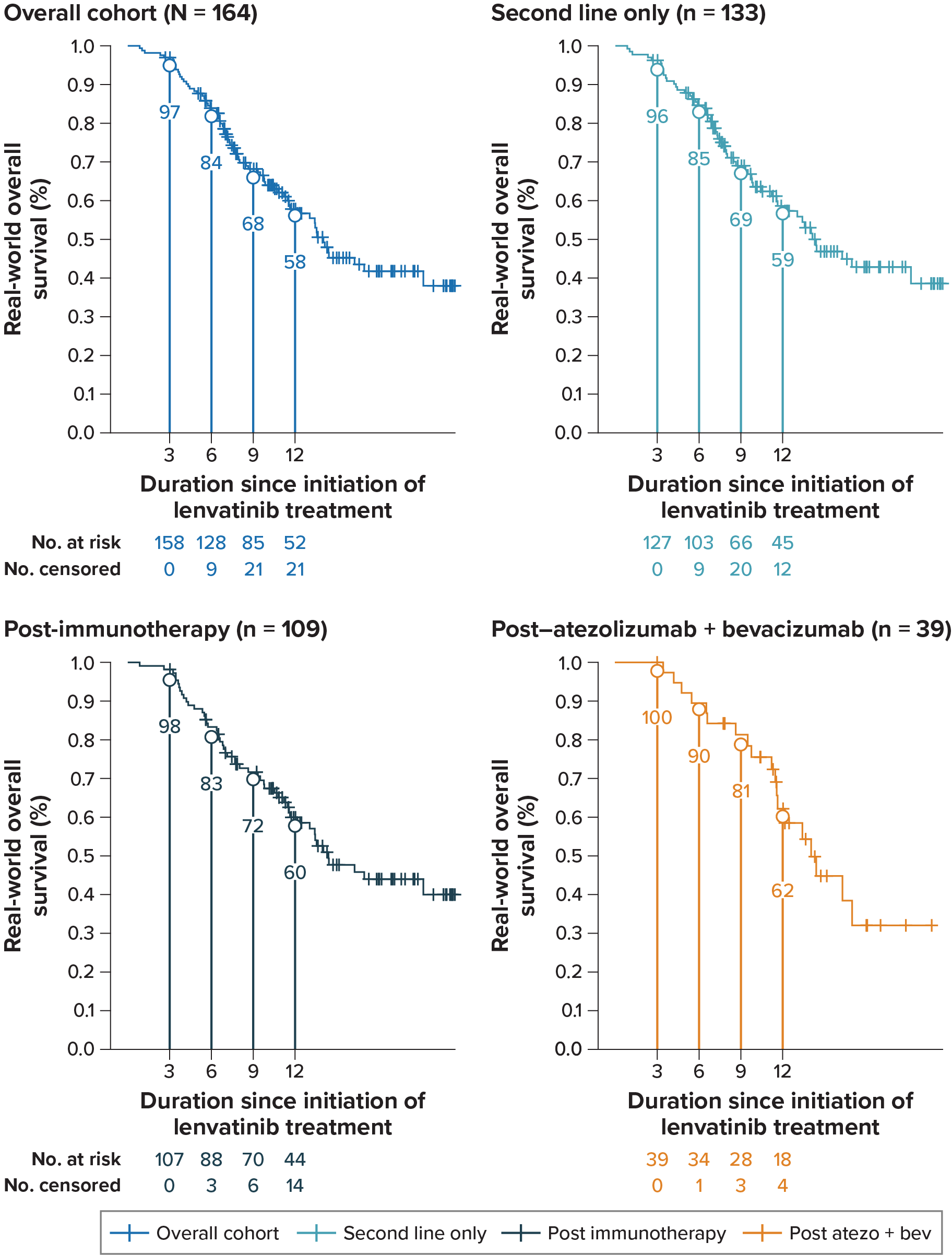

Supplement: Supplementary file 1 — Appendix S1: Supporting Information [file CNR2-6-e1679-s001.docx]
